# Development and validation of a nomogram to predict the risk of type II endoleak after endovascular aneurysm repair

**DOI:** 10.3389/fcvm.2025.1639697

**Published:** 2025-09-10

**Authors:** Bowen Liu, Xiaobin Tang, Nan He, Zhong Chen

**Affiliations:** Department of Vascular Surgery, Beijing Anzhen Hospital, Capital Medical University, Beijing, China

**Keywords:** type II endoleak, endovascular aneurysm repair, nomogram, risk prediction, aneurysm

## Abstract

**Objective:**

Type II endoleak (T2EL) is the most common complication following endovascular aneurysm repair (EVAR) of abdominal aortic aneurysms (AAA). T2EL may lead to aneurysm sac expansion and rupture. Identifying high-risk patients is crucial for prophylaxis and early intervention.

**Methods:**

This single-center retrospective study included 332 patients who underwent EVAR for infrarenal AAA. Demographic, clinical, anatomical, and medication-related data were collected. A nomogram was developed based on significant predictors. Its performance was assessed by receiver operating characteristic (ROC) curves, calibration plots, and decision curve analysis (DCA).

**Results:**

T2EL occurred in 70 (21.08%) of 332 patients. Multivariate logistic regression revealed six independent predictors: age, smoking status, intraluminal thrombus (ILT), number of patent lumbar arteries (LA), inferior mesenteric artery (IMA) diameter, and IMA patency. The nomogram demonstrated excellent calibration and strong predictive ability, with an area under the curve (AUC) of 0.806 (training set) and 0.758 (validation set). DCA showed clinical benefit across threshold probabilities of 1%–66% and 79%–92% in the training set, and 1%–84% in the validation set.

**Conclusion:**

The proposed nomogram effectively integrates clinical and anatomical factors to assess the risk of T2EL after EVAR. It may help identify patients requiring intensified surveillance or early interventions to mitigate complications. Further multicenter, prospective studies are needed to validate the nomogram's applicability.

## Introduction

Abdominal aortic aneurysm (AAA) is a life-threatening vascular disorder characterized by a localized dilation of the abdominal aorta, typically exceeding 50% of its normal diameter (1, 2). The widespread use of endovascular aneurysm repair (EVAR) has markedly improved the early postoperative outcomes for AAA compared with open repair, offering reduced perioperative morbidity and mortality, as well as shorter hospitalization (3, 4). However, despite these advantages, EVAR is associated with unique complications, most notably endoleaks, which can compromise the long-term success of the procedure.

Among the various types of endoleaks, Type II endoleak (T2EL), defined as retrograde blood flow into the aneurysm sac through patent collateral arteries such as the inferior mesenteric artery (IMA) or lumbar arteries (LA), remains the most common (5). Although T2ELs are often considered less immediately dangerous than Type I or Type III endoleaks, they still pose a significant risk for continued pressurization and eventual expansion of the aneurysm sac (6, 7). Some T2ELs close spontaneously within the first year, whereas others persist and lead to sac enlargement, thereby increasing the risk of rupture and necessitating secondary interventions (7).

Multiple factors have been associated with T2EL occurrence, including an enlarged IMA diameter, a higher number of patent lumbar arteries, advanced age, and certain anatomic features such as low ILT volume (8, 9). Yet, predicting which patients are at greatest risk of developing T2EL remains challenging. Early and accurate risk stratification would not only facilitate targeted surveillance strategies but also support more effective decision-making regarding preoperative or perioperative interventions, such as selective embolization of high-risk side branches.

As a graphical visualization of a statistical predictive model, nomogram has emerged as a powerful tool in individualized risk assessment for a variety of clinical conditions. Nomograms can incorporate multiple patient- and aneurysm-specific factors into a single, user-friendly interface, allowing clinicians to quickly estimate a patient's probability of an event (10). To date, however, few studies have focused on developing and validating nomograms specifically designed to predict T2EL after EVAR (11).

In this study, we sought to develop and validate a nomogram that can accurately predict the probability of T2EL in patients undergoing EVAR. Our model aims to integrate preoperative clinical variables, imaging parameters, and procedural factors to guide clinicians in identifying high-risk cases, optimizing perioperative strategies, and ultimately improving long-term outcomes for AAA patients.

## Methods

### Patients

In compliance with the Declaration of Helsinki, this single-center study was approved by the Institutional Review Committee of Beijing Anzhen Hospital (Approval No. 2025028x). Due to the retrospective retrieval of the patient's data, the requirement for informed consent was waived. Data retrieved from the hospital's medical data intelligence platform were anonymized to ensure patient privacy. We consecutively enrolled patients diagnosed with infrarenal AAA in the department of cardiovascular surgery at our institution between March 2018 and September 2023. All relevant medical records were obtained from the hospital's electronic health record system. All EVAR procedures were performed using a modular bifurcated infrarenal endograft (Medtronic endurant); no other graft types were used during the study period.

### T2EL diagnosis and follow-up

After EVAR, patients underwent routine imaging follow-up using duplex ultrasound and/or contrast-enhanced CT angiography at 1, 6, and 12 months postoperatively. These examinations were used to assess patient outcomes and detect the presence of endoleaks. T2EL was defined as persistent blood flow into the aneurysm sac through collateral arteries, such as the IMA or LA, occurring within the 1 year after treatment. Based on these imaging findings, patients were allocated to two groups: those who developed T2EL and those who did not.

### Inclusion and exclusion criteria

Eligible patients met the following criteria: (1) age ≥18 years, (2) a confirmed diagnosis of infrarenal AAA (maximum infrarenal aortic diameter ≥30 mm), and (3) EVAR as the primary repair. Patients were excluded if they (1) had previously undergone open surgical repair; (2) were in the non-T2EL group but lacked imaging follow-up extending to 12 months; (3) had insufficient medical data; (4) presented with a ruptured AAA; or (5) received prophylactic embolisation of the IMA or lumbar arteries at the time of EVAR.

### Variables

The clinical data regarding the patient's first visit to our hospital or their first admission were recorded. All variables were grouped into three main categories. Demographic and clinical data included age, body mass index (BMI), sex, smoking, drinking, hypertension, hyperlipidemia, diabetes, chronic obstructive pulmonary disease (COPD), coronary heart disease (CHD), chronic renal insufficiency, family history, and platelet levels. Aneurysm characteristics included neck angles (*α*° and *β*°), body maximum diameter, aneurysm body volume, number of patent LA, IMA diameter, aneurysm shape (fusiform/saccular), the presence or absence of ILT, and patency of the IMA. Medication usage covered anticoagulation, antiplatelet therapy, and statin use.

### Statistical analysis

Continuous variables were expressed as mean ± standard deviation $\left(\bar{x}\pm s\right)$ and compared between groups using the student's *t*-test. Categorical variables were presented as counts (*n*) and percentages (%) and analyzed using the *χ*^2^ test or Fisher's exact test, as appropriate. Multivariate logistic regression analysis was performed to identify potential risk factors for T2EL, and a nomogram was constructed to visualize the regression results. To further control for measured confounding in the smoking-T2EL comparison, we generated inverse-probability-weighted doubly-robust (IPTW-DR) based on a non-parsimonious propensity-score (PS) model. The PS was estimated for every patient with a logistic-regression model that included all variables. Each smoker was then weighted by 1/PS, whereas each non-smoker was weighted by 1/(1-PS). To improve numerical stability, weights were stabilized by multiplying by the marginal probability of the observed exposure and were truncated at the 1st and 99th percentiles. The effect of smoking on T2EL was then estimated with a weighted logistic-regression model that included the same set of covariates used to construct nomogram. The receiver operating characteristic (ROC) curve and the area under the curve (AUC) were used to assess the diagnostic performance of the nomogram. The Youden index, defined as sensitivity plus specificity minus one, was employed to determine the optimal sensitivity and specificity by identifying the maximum Youden index. Additionally, a calibration curve and decision curve analysis (DCA) were utilized to evaluate the nomogram's goodness of fit and clinical utility, respectively. All statistical analyses were conducted using SPSS software (version 27.0, IBM, Armonk, NY, USA) and R software (version 4.4.2, The R Foundation for Statistical Computing, Vienna, Austria). The R packages utilized in the analysis included “glmnet,” “RMS,” “Foreign,” “caret,” “rmda,” etc. All statistical tests were two-sided, and a *P*-value <0.05 was considered statistically significant.

## Results

### Patients

A total of 368 patients were initially enrolled, of whom 332 (90.22%) met the inclusion and exclusion criteria. Among them, 262 (78.92%) patients did not develop T2EL, while 70 patients (21.08%) were diagnosed with T2EL. To ensure the proper construction and validation of the model, the patients were randomly assigned into two groups based on the following proportions: 70% in the training set and 30% in the validation set. After randomization, 236 patients were allocated to the training set, while 96 patients were assigned to the validation set (Supplementary Figure S1). A comparison was conducted between the two groups regarding clinical characteristics, medical history, and aneurysm-related parameters. No significant differences were observed between the training and validation sets, indicating that the data distribution was consistent across both groups. This consistency ensures that the predictive model built from the training set can better reflect its true performance during validation, thereby enhancing its robustness and generalizability (Supplementary Table S1).

### Exploration of factors influencing T2EL occurrence

In the training set, a comparative analysis was conducted between patients with and without T2EL to evaluate potential influencing factors, including general clinical characteristics, medical history, and detailed aneurysm-related parameters. The results demonstrated that patients in the T2EL group had significantly higher age (67.9 vs. 64.62 years, *P* = 0.024), anticoagulation use rate (26.92% vs. 14.67%, *P* = 0.04), number of patent lumbar arteries (LA) (4.1 vs. 3.51, *P* = 0.009), IMA diameter (3.98 vs. 3.42 mm, *P* = 0.002), prevalence of ILT (42.31% vs. 14.13%, *P* < 0.001), and patency IMA rate (90.38% vs. 73.37%, *P* = 0.01) compared to the non-T2EL group. Conversely, the frequency of smoking was significantly lower in the T2EL group than in the non-T2EL group (51.92% vs. 70.65%, *P* = 0.012) (Table 1). Further multivariate logistic regression analysis identified age, smoking status, ILT, number of patent LA, IMA diameter, and patency IMA as independent predictors of T2EL occurrence, while anticoagulation use was not significantly associated with T2EL development (Table 2). To probe the unexpected inverse association between smoking and T2EL, we performed stratified multivariable logistic models and an IPTW-DR analysis. Stratified multivariable models confirmed that smoking retained an inverse but imprecise association with T2EL in both the IMA-patent (OR: 0.50, 95% CI: 0.24–1.07) and IMA-occluded (OR: 0.19, 95% CI: 0.02–2.01) strata, as well as in ILT-negative (OR: 0.41, 95% CI: 0.17–0.97) and ILT-positive (OR: 0.41, 95% CI: 0.10–1.64) subgroups. After IPTW-DR adjustment, the effect remained significance (OR: 0.105, 95% CI: 0.064–0.174, *P* < 0.001). Collectively, these findings suggest that the apparent “protective” effect of smoking likely reflects residual or collider confounding rather than a true causal relationship (Supplementary Table S2).

**Table 1:** Preoperative patient characteristics and anatomic variables for the training cohort and divided by groups.

Variables	Without T2EL (*n* = 184)	With T2EL (*n* = 52)	*χ* ^2^ */t*	*P*
	$\bar{x}\pm s$/*n* (%)	$\bar{x}\pm s$/*n* (%)		
Age	64.62 ± 9.34	67.90 ± 8.64	−2.27	**0** **.** **024**
BMI	26.45 ± 4.28	26.58 ± 4.20	−0.2	0.845
Sex			0.17	0.682
Male	73 (39.67)	19 (36.54)		
Female	111 (60.33)	33 (63.46)		
Smoking			6.39	**0** **.** **012**
No	54 (29.35)	25 (48.08)		
Yes	130 (70.65)	27 (51.92)		
Drinking			2.48	0.116
No	134 (72.83)	32 (61.54)		
Yes	50 (27.17)	20 (38.46)		
Hypertension			3.61	0.057
No	80 (43.48)	15 (28.85)		
Yes	104 (56.52)	37 (71.15)		
Hyperlipidemia			0.12	0.731
No	80 (43.48)	24 (46.15)		
Yes	104 (56.52)	28 (53.85)		
Diabetes			0.25	0.619
No	144 (78.26)	39 (75.00)		
Yes	40 (21.74)	13 (25.00)		
COPD			0.11	0.741
No	159 (86.41)	44 (84.62)		
Yes	25 (13.59)	8 (15.38)		
CHD			0.12	0.732
No	149 (80.98)	41 (78.85)		
Yes	35 (19.02)	11 (21.15)		
Chronic renal insufficiency			0.24	0.622
No	167 (90.76)	46 (88.46)		
Yes	17 (9.24)	6 (11.54)		
Family History			3.46	0.063
No	166 (90.22)	42 (80.77)		
Yes	18 (9.78)	10 (19.23)		
Anticoagulation			4.24	**0** **.** **04**
No	157 (85.33)	38 (73.08)		
Yes	27 (14.67)	14 (26.92)		
Neck angle (*α*°)	145.42 ± 7.86	146.79 ± 8.05	−1.1	0.273
Neck angle (*β*°)	153.89 ± 10.12	157.00 ± 10.13	−1.95	0.052
Body Maximum Diameter	5.73 ± 0.83	5.82 ± 0.97	−0.66	0.508
Diameter	2.73 ± 0.37	2.76 ± 0.38	−0.51	0.611
Aneurysm body volume	165.17 ± 74.09	182.07 ± 74.88	−1.45	0.149
Number of patent LA	3.51 ± 1.64	4.10 ± 1.35	−2.66	**0** **.** **009**
IMA diameter	3.42 ± 1.14	3.98 ± 1.10	−3.14	**0** **.** **002**
Platelets			2.48	0.116
Normal	50 (27.17)	20 (38.46)		
Abnormal	134 (72.83)	32 (61.54)		
Shape of aneurysm			0.27	0.607
Fusiform	150 (81.52)	44 (84.62)		
Saccular	34 (18.48)	8 (15.38)		
ILT			19.87	**<** **.** **001**
Absent	158 (85.87)	30 (57.69)		
Present	26 (14.13)	22 (42.31)		
Patency IMA			6.65	**0** **.** **01**
No	49 (26.63)	5 (9.62)		
Yes	135 (73.37)	47 (90.38)		
Antiplatelet therapy			0.02	0.902
No	26 (14.13)	7 (13.46)		
Yes	158 (85.87)	45 (86.54)		
Statin use			1.68	0.196
No	91 (49.46)	31 (59.62)		
Yes	93 (50.54)	21 (40.38)		

CHD, coronary-heart-disease; COPD, chronic obstructive pulmonary disease; IMA, inferior mesenteric artery; BMI, body mass index; LA, lumbar arteries; T2EL, type II endoleak; ILT, intraluminal thrombus.

The values in bold indicate statistically significant differences (*P* < 0.05).

**Table 2:** Multivariate logistic regression analysis of factors influencing T2EL.

Variables	*B*	Se	Wald	*P*	OR	95% CI of OR	
Age (year)	0.058	0.022	6.838	0.009	1.06	1.015	1.107
Smoking (Yes vs. No)	−0.801	0.368	4.726	0.03	0.449	0.218	0.924
ILT (Yes vs. No)	1.628	0.408	15.947	<0.001	5.095	2.291	11.328
Number of patent LA (*n*)	0.343	0.116	8.669	0.003	1.409	1.121	1.77
IMA diameter (mm)	0.334	0.168	3.964	0.046	1.396	1.005	1.939
Patency IMA (Yes vs. No)	1.214	0.54	5.046	0.025	3.365	1.167	9.702
Anticoagulation (Yes vs. No)	0.547	0.446	1.502	0.22	1.727	0.721	4.139
Intercept	−8.74	1.904	21.073	<0.001			

IMA, inferior mesenteric artery; LA, lumbar arteries; T2EL, type II endoleak; ILT, intraluminal thrombus.

### Establishment of the nomogram

**Figure 1:**
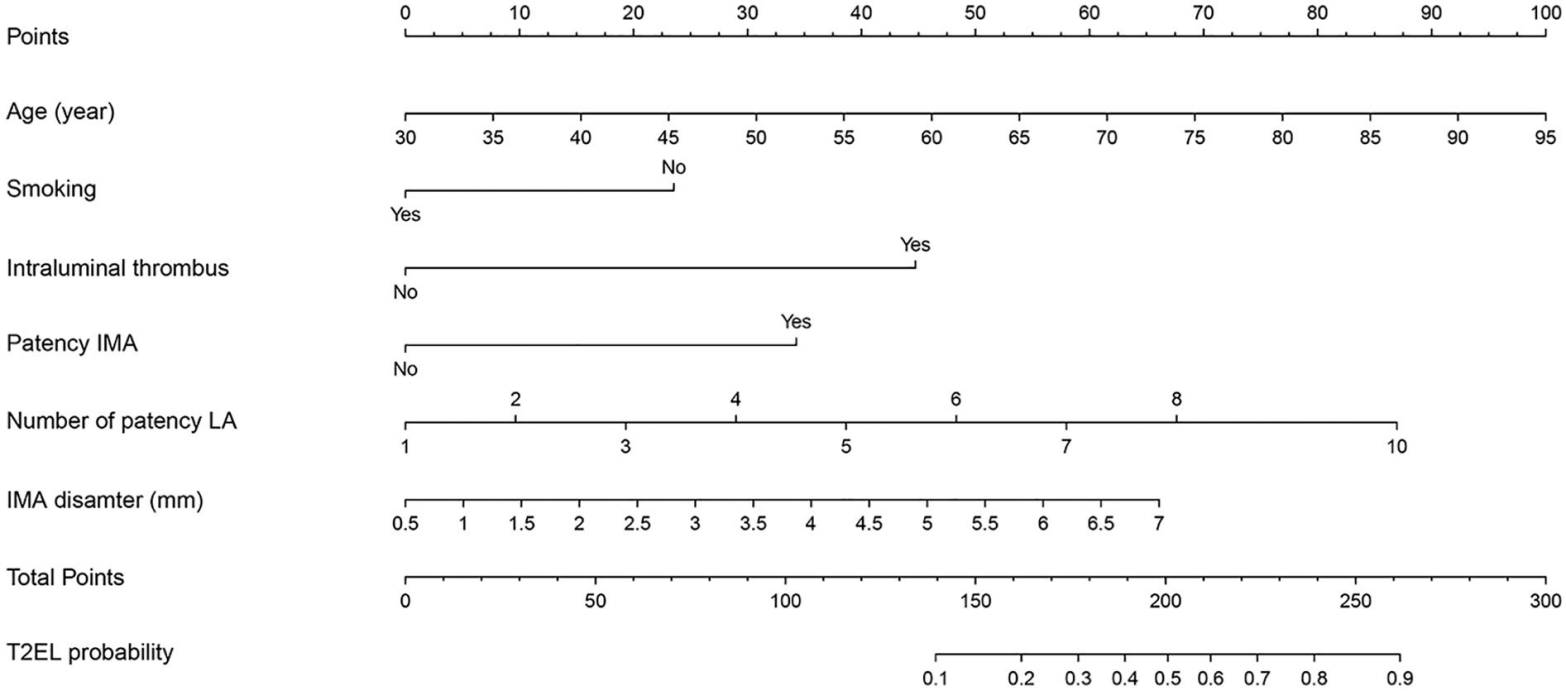
For estimating the probability of type II endoleak (T2EL) following endovascular aneurysm repair (EVAR). The nomogram incorporates six predictors including age, smoking status, intraluminal thrombus, number of patent lumbar arteries (LA), inferior mesenteric artery (IMA) diameter, and IMA patency. T2EL, type II endoleak; EVAR, endovascular aneurysm repair; LA, lumbar arteries; IMA, inferior mesenteric artery.

Based on the identified independent risk factors, a predictive nomogram was developed to estimate the likelihood of T2EL occurrence following EVAR. The model incorporated six variables: age, smoking status, presence of ILT, number of patent LA, IMA diameter, and patency of the IMA. The results demonstrated that all six factors were significantly associated with T2EL occurrence, with the following HR and 95% CI: Age (HR = 1.046, 95% CI: 1.009–1.084, *P* = 0.015), Smoking (HR = 0.433, 95% CI: 0.233–0.804, *P* = 0.008), ILT (HR = 5.243, 95% CI: 2.680–10.256, *P* < 0.001), Number of patent LA (HR = 1.433, 95% CI: 1.182–1.739, *P* < 0.001), IMA diameter (HR = 1.450, 95% CI: 1.118–1.880, *P* = 0.005), and Patency of the IMA (HR = 3.452, 95% CI: 1.347–8.851, *P* = 0.010) (Supplementary Table S3 and Figure 1). The final predictive model was expressed as follows: $\mathrm{In}\left(\frac{P}{1-P}\right)\;=\;0.045\;\times$ $\mathrm{Age}\;-\;0.838X_{\mathrm{smoking}}\;+\;1.675X_{\mathrm{ILT}}\;+\;0.36\;\times \;\mathrm{Number}\;\mathrm{of}\;\mathrm{patent}$ $\mathrm{LA}+0.372\;\times \mathrm{IMA}\;\mathrm{diameter}+1.239X_{\mathrm{Patency}\;\mathrm{IMA}}-7.967$, where *P* represents the probability of T2EL occurrence.

### Validation of the nomogram

**Figure 2:**
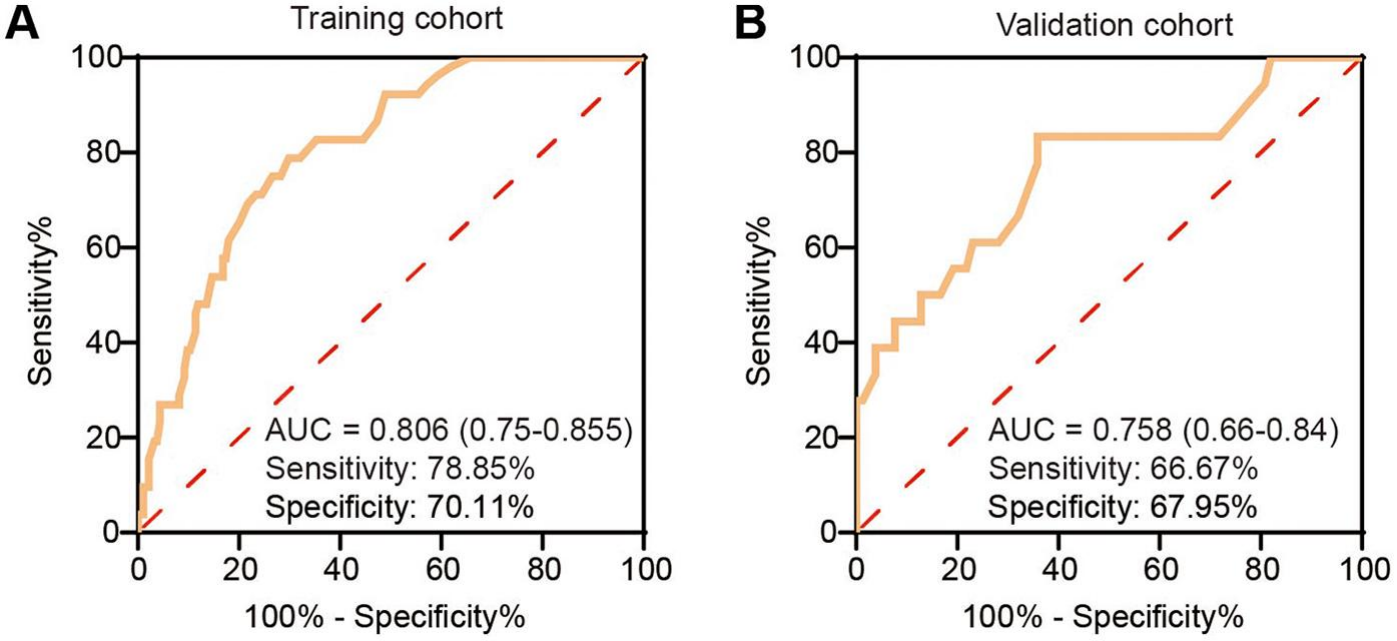
Calibration curves of the nomogram in the training and validation sets. **(A)** Calibration curve in the training set, showing strong agreement between predicted and observed probabilities (mean absolute error = 0.032, *B* = 1,000 bootstrap resamples). **(B)** Calibration curve in the validation set, also demonstrating good calibration (mean absolute error = 0.024, *B* = 1,000 bootstrap resamples). *n*, sample size; *B*, number of bootstrap iterations.

**Figure 3:**
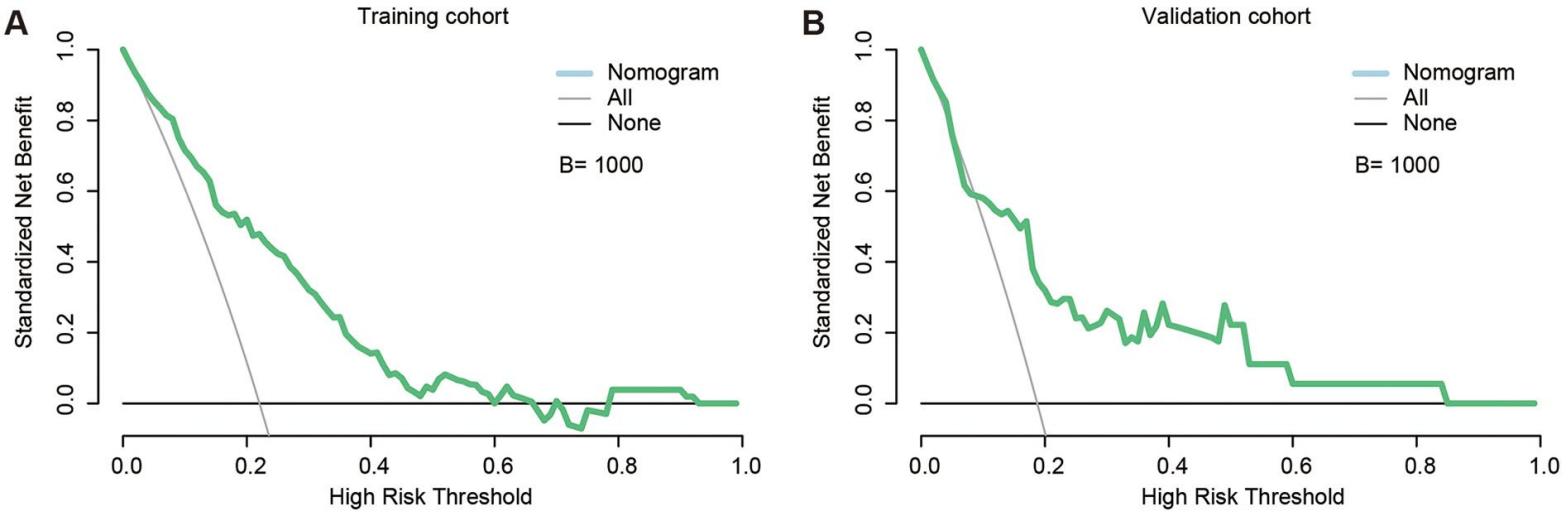
Receiver operating characteristic (ROC) curves of the nomogram predicting type II endoleak (T2EL) in the training and validation sets. **(A)** ROC curve in the training set. **(B)** ROC curve in the validation set. ROC, receiver operating characteristic; AUC, area under the curve; T2EL, type II endoleak.

The calibration curve of the nomogram for predicting TEL showed good agreement between the predicted and actual observed probability values (the mean absolute error = 0.032), and the same was observed in the validation set (the mean absolute error = 0.024, Figure 2). In addition, in the training set of the model, the *χ*^2^ of the Hosmer-Lemeshow test was 2.235 and the *P* value was 0.973. In the independent validation set of the model, the *χ*^2^ was 5.282 and the *P* value was 0.727, indicating that the nomogram had a good fitting degree and did not deviate from a perfect fit with the actual value. Moreover, the predictive model demonstrated strong discriminative ability in both the training and validation cohorts. In the training cohort, the nomogram achieved an AUC of 0.806 (95% CI: 0.750–0.855), with an optimal probability cutoff of 0.20, yielding a sensitivity of 78.9% and specificity of 70.1%. To account for potential optimism, we also conducted internal bootstrap validation (*B* = 1,000) within the training set, yielding an optimism-corrected AUC of 0.777 and calibration slope of 0.962. In addition, using this same 0.20 cutoff in the validation cohort, the nomogram maintained roust performance, with an AUC of 0.758 (95% CI: 0.667–0.795), a sensitivity of 66.7%, and a specificity of 67.9% (Figure 3). These results indicate that the model possesses high predictive accuracy and generalizability, further supporting its clinical applicability.

### Clinical practicability of the nomogram

**Figure 4:**
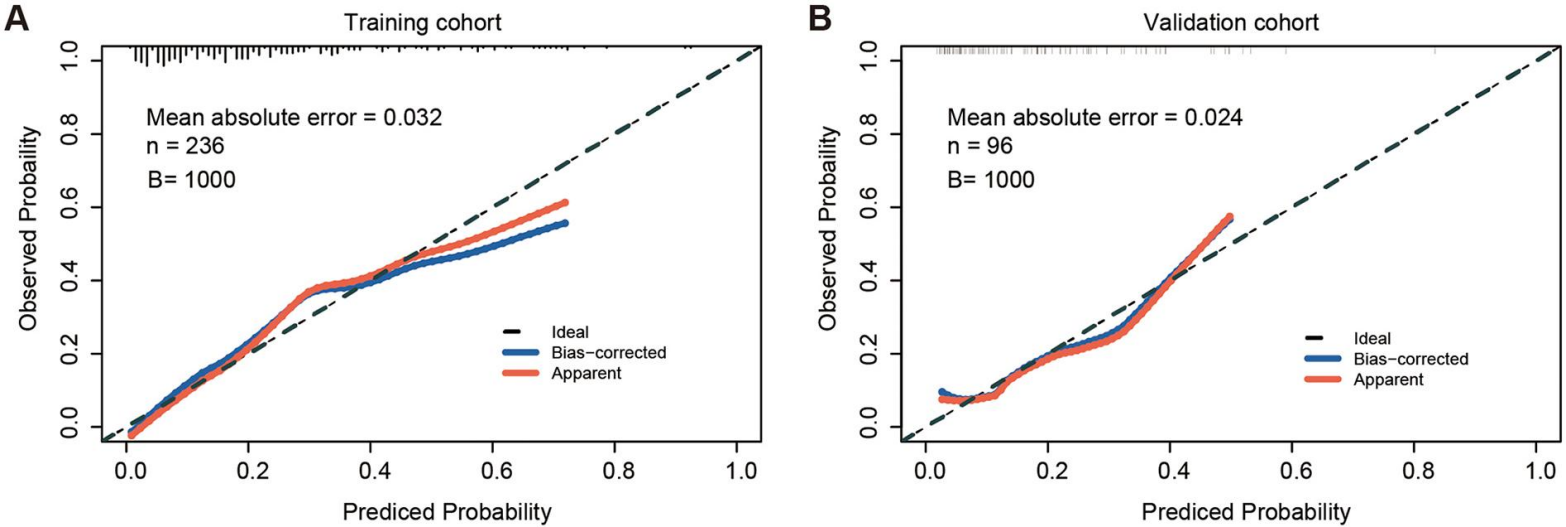
Decision curve analysis (DCA) of the nomogram. **(A)** DCA curve in the training set, demonstrating positive net clinical benefit across threshold probabilities of 1%–66% and 79%–92% (*B* = 1,000 bootstrap resamples). **(B)** DCA curve in the validation set, showing benefit across a broader threshold range of 1%–84% (*B* = 1,000 bootstrap resamples). DCA, decision curve analysis; *B*, number of bootstrap iterations.

Decision-curve analysis confirmed that the nomogram yields positive net benefit (NB) across a broad range of thresholds in both cohorts (Figure 4). In the training set, NB remained above zero from 1% to 66% and again from 79% to 92%. More importantly, at the thresholds most often used in daily practice the model provided substantial additional value: NB = 0.188 at 5%, 0.158 at 10%, 0.114 at 20%, 0.070 at 30%, and 0.030 at 40% (Supplementary Table S4). The validation set displayed the same monotonic pattern, with NB = 0.142, 0.109, 0.060, 0.049, and 0.042 at the corresponding cut-points. These results indicate that adopting a 10%–20% threshold would correctly manage 6–16 additional patients per 100, relative to treat-all or treat-none strategies, whereas a 20% cut-off offers a pragmatic balance between missed leaks and unnecessary interventions. Collectively, these findings support the nomogram's practical utility for personalised post-EVAR surveillance and selective prophylactic embolisation.

## Discussion

The present study aimed to identify key risk factors associated with T2EL following EVAR and to construct a practical nomogram for clinical decision-making. Our findings indicate that T2EL occurred in 70 (21.08%) of patients, a proportion that aligns with the generally reported incidence of 10%–30% in previous literature (8, 12–14). Six independent predictors, age, smoking status, ILT, number of patent lumbar arteries, IMA diameter, and patency of the IMA, emerged as central to T2EL development. Building on these predictors, the nomogram demonstrated robust predictive performance, featuring strong calibration and satisfactory discriminative power in both the training and validation cohorts. These results underscore the importance of integrating patient demographics, anatomical features, and procedural characteristics when assessing T2EL risk and highlight the potential role of a personalized risk stratification tool in optimizing post-EVAR surveillance and interventions.

Among anatomical factors, the number of patent LA and IMA diameter stood out in our analysis. This finding supports previous observations that a larger IMA diameter (>3 mm) and a higher count of patent LAs (≥4) significantly increase the risk of retrograde flow to the aneurysm sac, promoting T2EL (8, 11, 15). In addition, our data emphasize that IMA patency (i.e., absence of occlusion or embolization) further amplifies this risk, echoing studies where a patent IMA has been linked to persistent blood inflow that sustains or reinitiates a T2EL (15). The effect of ILT remains somewhat controversial across the literature (11, 16). While some groups propose that ILT can occlude side branches and thus reduce T2EL incidence (17), others, including our study, indicate a positive association between ILT and T2EL. One plausible explanation is that extensive or poorly organized thrombus can, paradoxically, trap arterial inflow within the aneurysm sac, perpetuating or exacerbating endoleaks (17). Hence, the role of ILT may depend on its location, volume, and extent, underscoring the complex hemodynamics at play in aneurysm sacs post-EVAR.

The most unexpected finding was an inverse association between smoking and T2EL in the primary model (adjusted OR: 0.48, 95% CI: 0.24–0.96), in line with two earlier series (16, 18). Mechanistically, nicotine-induced vasospasm, hypercoagulability and accelerated atherosclerosis might occlude lumbar arteries or the IMA and thus reduce retrograde sac perfusion (18). Although branch occlusion from smoking-related vasospasm or atherosclerosis might theoretically limit retrograde flow, stratified and IPTW-DR analyses still showed an inverse association. This statistically robust yet counter-intuitive result is best viewed as hypothesis-generating and probably reflects residual or collider confounding rather than a true protective effect; confirmation will require larger prospective studies with detailed imaging and competing-risk modelling. Regarding other comorbidities, we found age to be an influential parameter, which corroborates the notion that progressive aortic and collateral degeneration may predispose older patients to endoleak formation (11, 16). Meanwhile, variables such as anticoagulation, diabetes, and coronary heart disease were not significantly linked to T2EL in our model, possibly reflecting differences in patient cohorts, medication adherence, or endograft selection (16). Overall, these findings underscore the multifactorial nature of T2EL pathophysiology, highlighting the importance of both anatomic and clinical factors in predicting, monitoring, and managing endoleaks following EVAR.

Our nomogram exhibited excellent predictive strength, achieving an AUC of 0.806 in the training set and 0.758 in the validation set. Liu et al. (11) reported a slightly higher C-index of 0.92 for their four-item nomogram (age, smoking, IMA diameter, number of patent lumbar arteries), designed specifically to predict T2EL-related re-intervention rather than initial T2EL occurrence; three of these predictors overlap with our model, whereas we additionally incorporated ILT and IMA patency to allow pre-operative risk stratification and surveillance optimization. Furthermore, the DCA revealed a positive net benefit across a wide threshold probability range, reinforcing the clinical practicality of using the nomogram to guide early intervention. This result resonates with the principle of personalized medicine, wherein individualized risk prediction enables clinicians to optimize post-EVAR monitoring and selectively intervene in those at highest risk (7, 16). From a clinical standpoint, these findings contribute to the ongoing debate over early intervention vs. conservative management of T2EL. While some guidelines recommend immediate treatment when aneurysm sac expansion surpasses 5 mm in 6 months (19, 20), others advocate watchful waiting for stable or spontaneously resolving endoleaks (12). By distinguishing patients with substantially elevated risk, our nomogram may help identify those who could benefit from a proactive approach, especially in the context of prophylactic measures such as IMA or LA embolization (16). Indeed, emerging studies indicate that selective embolization of large or patent vessels prior to EVAR can reduce T2EL rates and subsequent reinterventions (15), suggesting that early, targeted interventions could further refine outcomes in high-risk patients. Our nomogram can be seamlessly incorporated into clinical workflows: the score—based entirely on data already obtained from pre-operative CTA and routine assessment—can be auto-generated within an electronic health-record module or a web/mobile calculator. We propose a 20% risk threshold (the Youden-optimised cut-point) as a practical trigger for action, because it still confers positive net benefit and aligns with the level at which most centres intensify surveillance or undertake prophylactic branch embolisation. For patients above this threshold, targeted measures—such as embolisation of a patent IMA or dominant lumbar artery (>3 mm, minimal sac thrombus) or pre-emptive coiling during the index EVAR—may reduce subsequent type II endoleaks. Using the nomogram in this way shifts management toward a proactive, anatomy-driven, and personalised strategy while avoiding unnecessary interventions in lower-risk patients.

Despite the strengths of our investigation, several limitations warrant consideration. First, the single-center retrospective design may introduce selection bias and constrain the generalizability of our findings to broader populations or institutions with different patient demographics. Second, although we endeavored to include comprehensive variables, ranging from clinical and anatomical features to medication use, there remain potential unmeasured confounders, such as genetic predispositions and detailed biochemical markers, that could influence the development of T2EL. Third, although we conducted internal validation of the nomogram, external validation in a multicenter prospective cohort would further establish its reliability and practical utility. Fourth, all patients were treated with a single modular bifurcated infrarenal endograft, so the influence of different endograft designs or materials on T2EL risk could not be evaluated.

In conclusion, our study proposes and validates a nomogram that successfully integrates clinical, anatomical, and lifestyle factors to predict T2EL risk after EVAR. By pinpointing key contributors, including age, smoking status, ILT, number of patent lumbar arteries, IMA diameter, and patency of the IMA, this model demonstrates robust discrimination and calibration, and offers a valuable clinical tool for early intervention decision-making. While further prospective, multicenter research is needed to confirm its applicability in diverse patient populations, the present findings underscore the nomogram's potential to enhance personalized treatment strategies, reduce unnecessary reinterventions, and ultimately improve long-term outcomes for patients undergoing EVAR.

## Data Availability

The raw data supporting the conclusions of this article will be made available by the authors, without undue reservation.
