# Supplementary material for: Development and validation of a nomogram to predict the risk of type II endoleak after endovascular aneurysm repair
Source: Front Cardiovasc Med. 2025 Sep 10;12:1639697. doi: 10.3389/fcvm.2025.1639697 (PMC12457305; doi:10.3389/fcvm.2025.1639697)
Supplement: Supplementary file 1 [file Datasheet1.docx]

**Supplementary files**

**Article title:** Development and validation of a nomogram to predict the risk of type II endoleak after endovascular aneurysm repair

**Names and affiliations of authors**

Bowen Liu^1^, Xiaobin Tang^1^, Nan He^1^, Zhong Chen^1,*^

^1^Department of Vascular Surgery, Beijing Anzhen Hospital, Capital Medical University, Beijing, China.

**Corresponding author details:**

Zhong Chen. Email: vascularsurgeon1103@gmail.com.

Department of Vascular Surgery, Beijing Anzhen Hospital, Capital Medical University, Beijing, China.

**This file includes:**

Supplemental Figure 1

Supplemental Tables 1 to 4


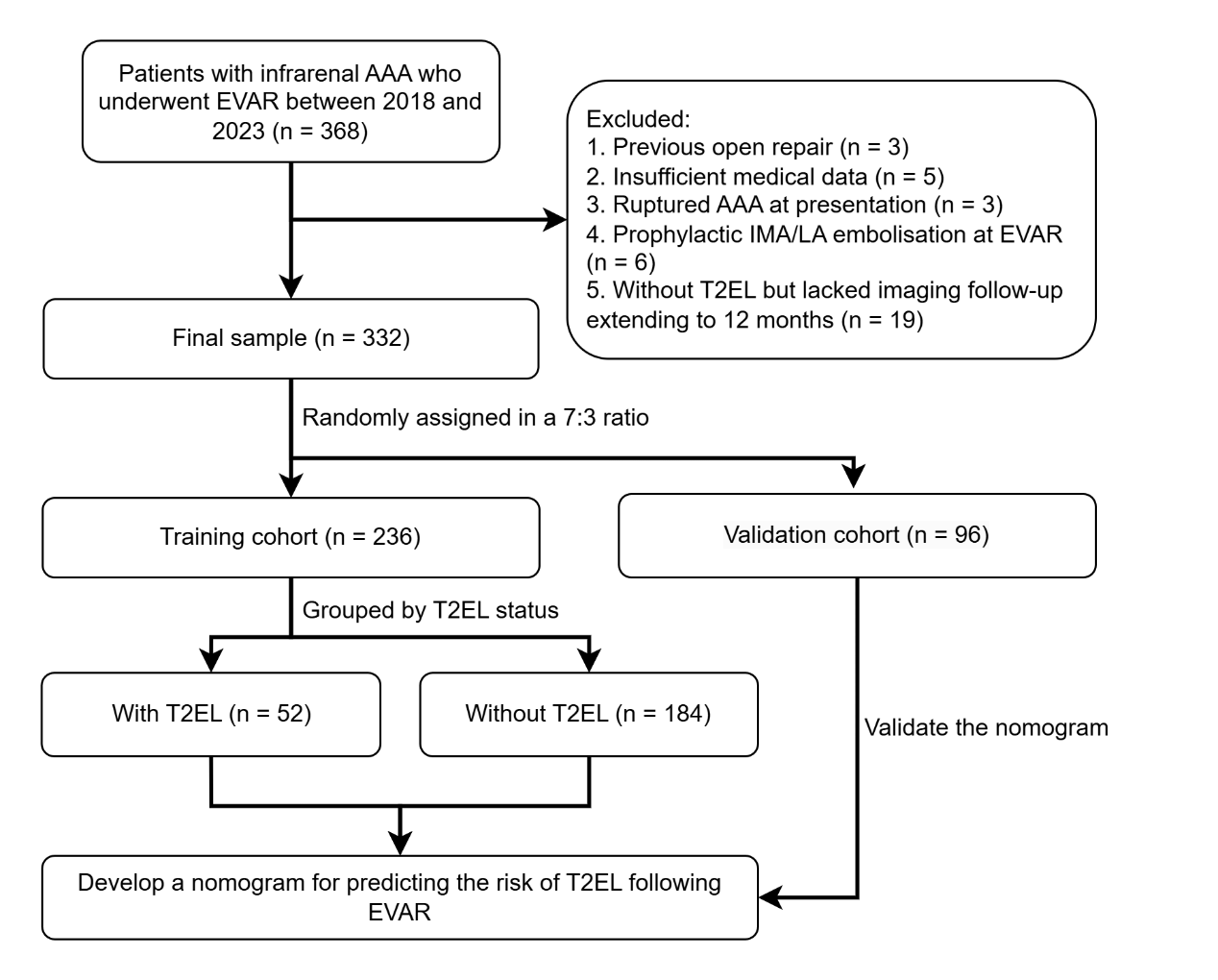
**Supplement Figure 1.** Flow chart Retrospective analysis process and patient exclusion criteria. *Abbreviations: T2EL,* *type II endoleak; EVAR, endovascular aneurysm repair; LA, lumbar arteries; IMA,* *inferior mesenteric artery.*

**Supplement Table 1: Patients’ demographic data and preoperative risk factors between training cohort and validation cohort.**

| Variables | Training cohort  (n = 236) | Validation cohort (n = 96) | Statistic | P |
| --- | --- | --- | --- | --- |
|  | $\bar{x}\pm s$/n(%) | $\bar{x}\pm s$/n(%) |  |  |
| Age | 65.34 ± 9.28 | 66.35 ± 8.22 | -0.93 | 0.353 |
| BMI | 26.48 ± 4.25 | 25.98 ± 4.60 | 0.94 | 0.35 |
| Sex |  |  | 1.32 | 0.25 |
| Male | 92 (38.98) | 44 (45.83) |  |  |
| Female | 144 (61.02) | 52 (54.17) |  |  |
| Smoking |  |  | 2.29 | 0.13 |
| No | 79 (33.47) | 24 (25.00) |  |  |
| Yes | 157 (66.53) | 72 (75.00) |  |  |
| Drinking |  |  | 0.08 | 0.775 |
| No | 166 (70.34) | 66 (68.75) |  |  |
| Yes | 70 (29.66) | 30 (31.25) |  |  |
| Hypertension |  |  | 3.6 | 0.058 |
| No | 95 (40.25) | 28 (29.17) |  |  |
| Yes | 141 (59.75) | 68 (70.83) |  |  |
| Hyperlipidemia |  |  | 0 | 0.958 |
| No | 104 (44.07) | 42 (43.75) |  |  |
| Yes | 132 (55.93) | 54 (56.25) |  |  |
| Diabetes |  |  | 0.01 | 0.908 |
| No | 183 (77.54) | 75 (78.12) |  |  |
| Yes | 53 (22.46) | 21 (21.88) |  |  |
| COPD |  |  | 0.38 | 0.538 |
| No | 203 (86.02) | 85 (88.54) |  |  |
| Yes | 33 (13.98) | 11 (11.46) |  |  |
| CHD |  |  | 1.24 | 0.265 |
| No | 190 (80.51) | 72 (75.00) |  |  |
| Yes | 46 (19.49) | 24 (25.00) |  |  |
| Chronic renal insufficiency | |  | 0.01 | 0.917 |
| No | 213 (90.25) | 87 (90.62) |  |  |
| Yes | 23 (9.75) | 9 (9.38) |  |  |
| Family History |  |  | 1.51 | 0.219 |
| No | 208 (88.14) | 89 (92.71) |  |  |
| Yes | 28 (11.86) | 7 (7.29) |  |  |
| Anticoagulation |  |  | 0.02 | 0.877 |
| No | 195 (82.63) | 80 (83.33) |  |  |
| Yes | 41 (17.37) | 16 (16.67) |  |  |
| Neck angle(α°) | 145.72 ± 7.91 | 144.58 ± 7.11 | 1.23 | 0.221 |
| Neck angle(β°) | 154.58 ± 10.19 | 153.49 ± 10.02 | 0.89 | 0.377 |
| Body Maximum Diameter | 5.75 ± 0.86 | 5.70 ± 0.74 | 0.44 | 0.658 |
| Diameter | 2.73 ± 0.37 | 2.77 ± 0.31 | -0.86 | 0.392 |
| Aneurysm body volume | 168.90 ± 74.44 | 167.02 ± 81.69 | 0.2 | 0.84 |
| Number of patency LA | 3.64 ± 1.59 | 3.48 ± 1.65 | 0.8 | 0.423 |
| IMA disamter | 3.55 ± 1.15 | 3.45 ± 1.25 | 0.71 | 0.475 |
| Platelets |  |  | 0.43 | 0.511 |
| Normal | 70 (29.66) | 32 (33.33) |  |  |
| Abnormal | 166 (70.34) | 64 (66.67) |  |  |
| Shape of aneurysm |  |  | 0.19 | 0.666 |
| Fusiform | 139 (58.90) | 59 (61.46) |  |  |
| Saccular | 97 (41.10) | 37 (38.54) |  |  |
| Intraluminal Thrombus | |  | 0.01 | 0.91 |
| Absent | 188 (79.66) | 77 (80.21) |  |  |
| Present | 48 (20.34) | 19 (19.79) |  |  |
| Patency IMA |  |  | 1.09 | 0.297 |
| No | 54 (22.88) | 17 (17.71) |  |  |
| Yes | 182 (77.12) | 79 (82.29) |  |  |
| Antiplatelet therapy | |  | 1.19 | 0.275 |
| No | 33 (13.98) | 18 (18.75) |  |  |
| Yes | 203 (86.02) | 78 (81.25) |  |  |
| Statin use |  |  | 0.63 | 0.426 |
| No | 122 (51.69) | 45 (46.88) |  |  |
| Yes | 114 (48.31) | 51 (53.12) |  |  |
| T2EL |  |  | 0.44 | 0.506 |
| Without | 184 (77.97) | 78 (81.25) |  |  |
| With | 52 (22.03) | 18 (18.75) |  |  |

*CHD* coronary-heart-disease, *COPD* chronic obstructive pulmonary disease, *IMA* inferior mesenteric artery, *BMI* body mass index, *LA* lumber arteries. *T2EL* type II endoleak.

**Supplement Table 2: Stratified and doubly-robust sensitivity analyses for the association between smoking and T2EL**

|  |  | *B* | *Se* | *Wald* | *OR* | *95%CI of OR* | | *P* |
| --- | --- | --- | --- | --- | --- | --- | --- | --- |
| Patency IMA (No) | Age | 0.11 | 0.112 | 0.958 | 1.116 | 0.896 | 1.39 | 0.328 |
|  | Smoking | -1.64 | 1.192 | 1.894 | 0.194 | 0.019 | 2.006 | 0.169 |
|  | intraluminal thrombus | 3.668 | 1.686 | 4.731 | 39.156 | 1.437 | 1066.802 | 0.03 |
|  | Number of patency lumber arteries | 1.143 | 0.669 | 2.914 | 3.135 | 0.844 | 11.64 | 0.088 |
|  | IMA disamter | 0.158 | 0.581 | 0.074 | 1.171 | 0.375 | 3.662 | 0.785 |
|  | Intercept | -15.435 | 10.095 | 2.338 | 0 |  |  | 0.126 |
| Patency IMA (Yes) | Age | 0.051 | 0.023 | 5.141 | 1.053 | 1.007 | 1.1 | 0.023 |
|  | Smoking | -0.685 | 0.385 | 3.164 | 0.504 | 0.237 | 1.072 | 0.075 |
|  | intraluminal thrombus | 1.381 | 0.433 | 10.154 | 3.978 | 1.702 | 9.302 | 0.001 |
|  | Number of patency lumber arteries | 0.312 | 0.119 | 6.88 | 1.366 | 1.082 | 1.725 | 0.009 |
|  | IMA disamter | 0.411 | 0.183 | 5.029 | 1.509 | 1.053 | 2.162 | 0.025 |
|  | Intercept | -7.113 | 1.846 | 14.845 | 0.001 |  |  | 0 |
| Intraluminal thrombus (No) | Age | 0.064 | 0.025 | 6.349 | 1.066 | 1.014 | 1.121 | 0.012 |
|  | Smoking | -0.901 | 0.442 | 4.145 | 0.406 | 0.171 | 0.967 | 0.042 |
|  | Number of patency lumber arteries | 0.387 | 0.133 | 8.495 | 1.472 | 1.135 | 1.909 | 0.004 |
|  | IMA disamter | 0.374 | 0.21 | 3.182 | 1.454 | 0.964 | 2.193 | 0.074 |
|  | Patency IMA | 1.737 | 0.799 | 4.727 | 5.682 | 1.187 | 27.206 | 0.03 |
|  | Intercept | -9.77 | 2.345 | 17.357 | 0 |  |  | 0 |
| Intraluminal thrombus (Yes) | Age | 0.018 | 0.049 | 0.129 | 1.018 | 0.925 | 1.12 | 0.719 |
|  | Smoking | -0.893 | 0.709 | 1.586 | 0.409 | 0.102 | 1.644 | 0.208 |
|  | Number of patency lumber arteries | 0.219 | 0.255 | 0.738 | 1.245 | 0.755 | 2.051 | 0.39 |
|  | IMA disamter | 0.541 | 0.331 | 2.671 | 1.719 | 0.898 | 3.29 | 0.102 |
|  | Patency IMA | 0.431 | 0.835 | 0.267 | 1.539 | 0.3 | 7.909 | 0.605 |
|  | Intercept | -4.04 | 3.507 | 1.327 | 0.018 |  |  | 0.249 |
| IPTW-DR | Age | 0.072 | 0.015 | 22.196 | 1.074 | 1.043 | 1.107 | <0.001 |
|  | Smoking | -2.25 | 0.256 | 77.156 | 0.105 | 0.064 | 0.174 | <0.001 |
|  | intraluminal thrombus | 1.356 | 0.264 | 26.468 | 3.881 | 2.315 | 6.506 | <0.001 |
|  | Number of patency lumber arteries | 0.356 | 0.084 | 17.803 | 1.428 | 1.21 | 1.684 | <0.001 |
|  | IMA disamter | 0.745 | 0.129 | 33.492 | 2.107 | 1.637 | 2.712 | <0.001 |
|  | Patency IMA | 2.203 | 0.407 | 29.29 | 9.052 | 4.076 | 20.103 | <0.001 |
|  | Intercept | -12.007 | 1.428 | 70.741 | 0 |  |  | <0.001 |

*IMA* inferior mesenteric artery, *LA* lumber arteries, *T2EL* type II endoleak, *IPTW-DR* inverse-probability-weighted doubly-robust.

**Supplement Table 3: Multivariate logistic regression analysis of factors influencing T2EL**

|  | *B* | *Se* | *Wald* | *P* | *OR* | *95%CI of OR* | |
| --- | --- | --- | --- | --- | --- | --- | --- |
| Age (year) | 0.045 | 0.018 | 5.913 | 0.015 | 1.046 | 1.009 | 1.084 |
| Smoking (Yes vs No) | -0.838 | 0.316 | 7.024 | 0.008 | 0.433 | 0.233 | 0.804 |
| Intraluminal thrombus (Yes vs No) | 1.657 | 0.342 | 23.427 | <0.001 | 5.243 | 2.68 | 10.256 |
| Number of patency LA (n) | 0.36 | 0.099 | 13.359 | <0.001 | 1.433 | 1.182 | 1.739 |
| IMA disamter (mm) | 0.372 | 0.133 | 7.851 | 0.005 | 1.45 | 1.118 | 1.88 |
| Patency IMA (Yes vs No) | 1.239 | 0.48 | 6.653 | 0.01 | 3.452 | 1.347 | 8.851 |
| Intercept | -7.967 | 1.531 | 27.077 | <0.001 |  |  |  |

*IMA* inferior mesenteric artery, *LA* lumber arteries, *T2EL* type II endoleak.

**Supplement Table 4: The net benefit of decision curve analysis**

| Threshold | Net benefit | |
| --- | --- | --- |
|  | Training cohort | Validation cohort |
| 5% | 0.188 | 0.142 |
| 10% | 0.158 | 0.109 |
| 20% (ROC cut-off) | 0.114 | 0.060 |
| 30% | 0.070 | 0.049 |
| 40% | 0.030 | 0.042 |
| 50% | 0.008 | 0.042 |
| 60% | 0.000 | 0.010 |
| 70% | 0.001 | 0.010 |
| 80% | 0.008 | 0.010 |
| 90% | 0.000 | 0.000 |
